# Assessing data bias in visual surveys from a cetacean monitoring programme

**DOI:** 10.1038/s41597-022-01803-7

**Published:** 2022-11-10

**Authors:** Cláudia Oliveira-Rodrigues, Ana M. Correia, Raul Valente, Ágatha Gil, Miguel Gandra, Marcos Liberal, Massimiliano Rosso, Graham Pierce, Isabel Sousa-Pinto

**Affiliations:** 1 Coastal Biodiversity Laboratory, CIIMAR – Interdisciplinary Centre of Marine and Environmental Research, 4450-208 Matosinhos, Portugal; 2 Department of Biology, FCUP – Faculty of Sciences of the University of Porto, 4169-007 Porto, Portugal; 3 Department of Biology and Environment, CITAB – Centro de Investigação e Tecnologias Agroambientais e Biológicas, University of Trás-os-Montes and Alto Douro, 5000-801 Vila Real, Portugal; 4 CSIC – Consejo Superior de Investigaciones Científicas, 36208, Vigo, Pontevedra, Spain; 5 CCMAR – Centre of Marine Sciences, University of Algarve, Campus de Gambelas, 8005-139 Faro, Portugal; 6 Fraunhofer AICOS, 4200-135 Porto, Portugal; 7 CIMA Research Foundation – Centro Internazionale in Monitoraggio Ambientale, 17100 Savona, Italy; 8 Oceanlab, University of Aberdeen, AB41 6AA Aberdeen, United Kingdom

**Keywords:** Biogeography, Ecological modelling, Biodiversity, Data processing, Data publication and archiving

## Abstract

Long-term monitoring datasets are fundamental to understand physical and ecological responses to environmental changes, supporting management and conservation. The data should be reliable, with the sources of bias identified and quantified. CETUS Project is a cetacean monitoring programme in the Eastern North Atlantic, based on visual methods of data collection. This study aims to assess data quality and bias in the CETUS dataset, by 1) applying validation methods, through photographic confirmation of species identification; 2) creating data quality criteria to evaluate the observer’s experience; and 3) assessing bias to the number of sightings collected and to the success in species identification. Through photographic validation, the species identification of 10 sightings was corrected and a new species was added to the CETUS dataset. The number of sightings collected was biased by external factors, mostly by sampling effort but also by weather conditions. Ultimately, results highlight the importance of identifying and quantifying data bias, while also yielding guidelines for data collection and processing, relevant for species monitoring programmes based on visual methods.

## Introduction

Cetaceans play fundamental roles in aquatic ecosystems as umbrella and key species in the marine environment, good bioindicators, and sentinels of disturbances^1,2^. On top of that, they are charismatic, therefore ideal model species for education purposes and to encourage marine preservation and conservation^1,3^. Hence, cetaceans should be a priority in research for the management, conservation, and protection of the ocean. For the efficient protection of cetaceans, it is crucial to monitor their distribution and abundance, determine population size, and identify potential threats to their populations and habitats. Furthermore, it is fundamental to maintain long-term monitoring programmes to track changes over space and time, to investigate trends, and to increase forecasting capability, in order to capacitate policy-makers for the informed management of marine ecosystems^4–6^. For this purpose, the data collected has to be reliable, and it is desirable that it is made public, following the FAIR Guiding Principles, which enhances reusability by stakeholders: from the scientific community to decision-makers^7^. Moreover, not only the data should be made available and accessible, but the data quality should be known and quantified^8^.

**Fig. 1:**
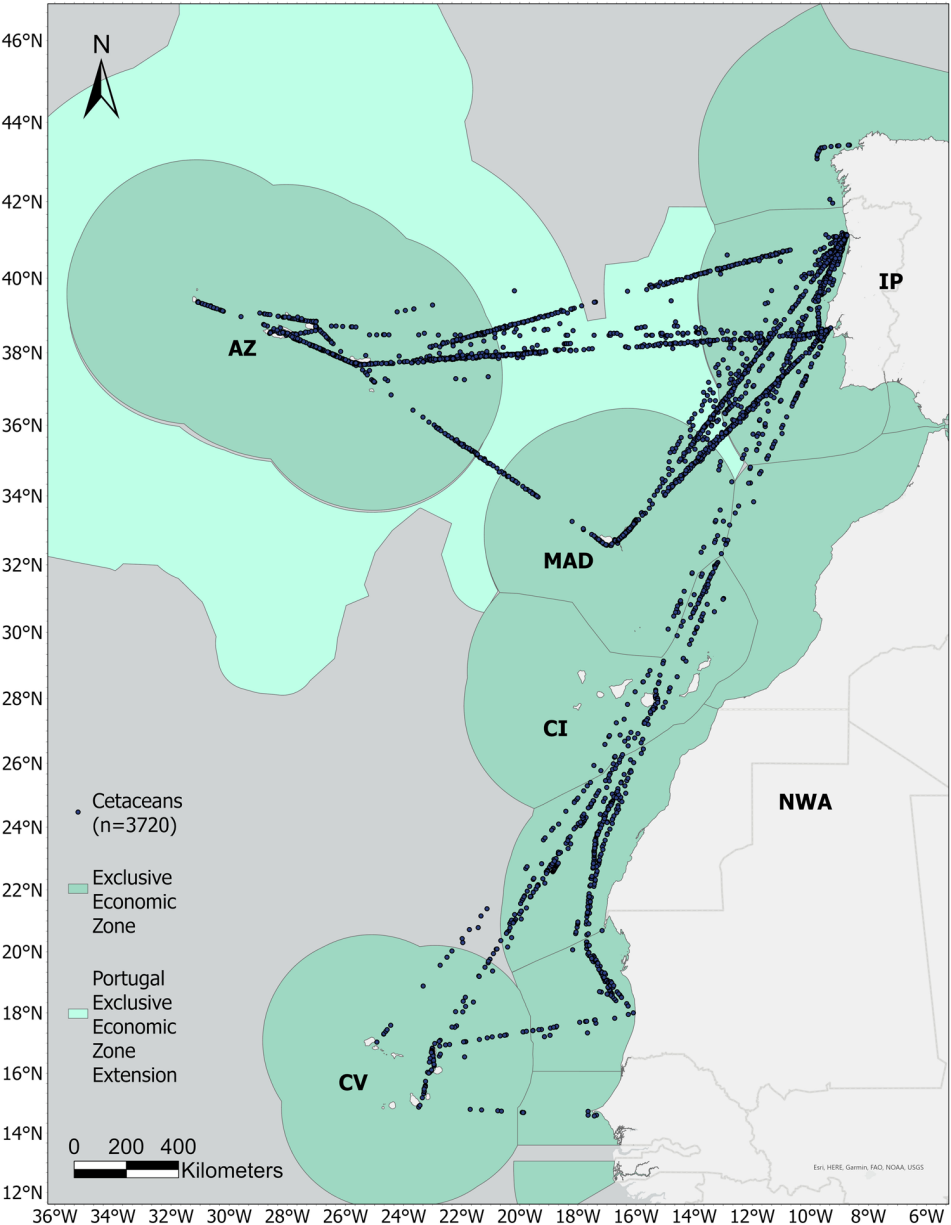
CETUS Project cetacean occurrences. CETUS study area with cetacean occurrences collected between 2012 and 2019. IP – Iberian Peninsula; NWA – Northwest Africa; AZ – Azores; MAD – Madeira; CI – Canary Islands; CV – Cape Verde.

CETUS Project is a long-term cetacean monitoring programme that started in 2012, to provide cetacean occurrence data in the vast region of the Eastern North Atlantic (ENA; Fig. 1). CETUS uses the cargo ships from TRANSINSULAR, a Portuguese company for maritime transport, as platforms of opportunity to collect data on their long-transect routes. In 2019, the first version of the CETUS dataset was made available open access at OBIS and EMODnet portals^9^ (updated version of the dataset^10^). The CETUS data verification and validation methods are rigorous, but they have been mostly related to data collection and processing, i.e.: i) embark of motivated, highly trained, and dedicated observers; ii) standard protocol for line-transect data collection; iii) recording of survey effort; iv) register of potential sources of bias during fieldwork; v) multiple verification steps in data entry and processing. None of these methods includes photographic verification/validation of the sightings or the assessment of data bias in terms of species detection and identification^9^.

Some cetacean species have similar morphology, making them hard to differentiate and leading to misidentifications, especially when sightings are distant from the monitoring lookout and the time available for observation and identification is short. This is often the case at sea^4,11^ and, therefore, verification and validation of photographic or video records are important tools to support species identification. Variability in the experience of the Marine Mammal Observers (MMO) is also a relevant bias to the data, and may impact on its quality^12,13^. Besides different capabilities in detecting and identifying species, MMOs experience with the sampling protocol and the environment of the survey is also determinant (e.g., sampling marine offshore areas aboard large vessels is substantially different from near-shore surveys aboard small vessels). These data bias are still largely disregarded in marine mammal occurrence datasets derived from visual surveys. Other factors that impact detectability, such as the height of the observation platform, and distance of sighting to the monitoring lookout point, are often corrected using correction factors or estimating the Effective Strip Width^14,15^. However, it is not always possible to employ such techniques^9^. Finally, weather conditions (e.g., sea state), are also important sources of bias, and often included when developing ecological niche modelling of cetaceans (based on visual records)^16–18^.

In 2022, a 2^nd^ version of the CETUS dataset was submitted (data from 2012–2017), including variables on data quality, namely the observer’s experience and the photographic validation of records^10^. Here, we present the methods applied in the update of the CETUS dataset with the aim to improve its informed usage, providing information on data quality and bias, by: i) applying new verification and validation methods based on the photographic/video confirmation of the species identifications; ii) creating quality criteria to evaluate the MMOs experience; and iii) modelling the influence of bias parameters on the sightings recorded per survey and on the identification success. The results were also used to identify useful improvements and to define guidelines for logistics and protocols of CETUS, and similar monitoring programmes, such as how to: i) improve the collection of photographic records for a better verification/validation process; ii) organize MMOs’ teams considering their experience; and iii) define meteorological thresholds for reliable sampling of cetacean occurrence/species identification.

## Results

### Photographic verification

In total, 9,970 photographic and video records of the CETUS sightings were collated. These records were obtained from 21 MMOs out of the 61 that participated in CETUS, and corresponded to 315 single-taxon sightings. From these 315 sightings photographed/videotaped, 287 (~91.1%) were matched with the CETUS dataset records, corresponding to ~7.7% of the total sightings in the dataset. The best match results correspond to the suborder Odontoceti (~9.2% records matched) (Table 1).

**Table 1:** Results of the photographic verification process.

Photographic Verification		
	M	NM
Odontoceti	254 (9.2%)	2522 (90.8%)
Mysticeti	20 (3.9%)	494 (96.1%)
NI	13 (3.0%)	417 (97.0%)
Total	287 (7.7%)	3433 (92.3%)

Number and percentage of the matched (M) and non-matched (NM) sightings, with photo/video records. The percentage is in relation to the total number of sightings recorded in the dataset, by suborder, non-identified (NI) and total cetacean occurrences.

### Photographic validation

Out of all of the matched records (total of 287), 250 were validated to the taxon registered in the dataset (37 photo/video records not validated, due to, e.g., photo quality, distance of animal(s), light, angle). A total of 170 photographs/videos (~59.2%) allowed for an identification up to the species level – complete validation.

In total, 10 occurrence records were originally registered with a misidentified species by the MMOs (~5.9% of the 170 validated records, to species level). From these, there were 9 misidentifications between delphinid species, of which 4 were within *Stenella* genus. As a result of the validation process, it was possible to reach a lower taxon (in comparison to the one originally recorded by the MMOs) in 49 sightings.

In the updated dataset (2^nd^ version), the sightings are now corrected according to the validation process, with 223 records validated to the taxon level they are registered with, plus 27 validated to the family. In Supplementary Table S1, the updated number of cetacean occurrences is displayed, which includes 3 sightings of a new species to the CETUS dataset: *Mesoplodon europaeus*.

### Creating a data quality criteria: evaluating MMOs experience

**Fig. 2:**
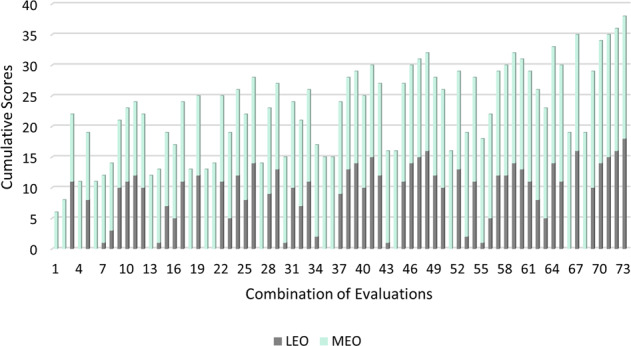
Evaluation scores of the MMO’s teams. Cumulative scores (sum of the evaluation scores of the MMOs), obtained for each combination of evaluations for the Most Experienced Observer (MEO) and the Least Experienced Observer (LEO). Evaluation scores were determined from the experience of the Marine Mammal Observers (MMOs), according to the created criteria (see methods).

The experience of 61 MMOs was evaluated following the created criteria, through 80 *curricula vitae* (due to alumni MMOs, see methods). This resulted in evaluation scores for each member of the MMOs team on board each cruise, generating an evaluation for the Most Experienced Observer (MEO) and Least Experienced Observer (LEO) - usually teams of two MMOs on board (see methods). Out of the 564 cruises monitored, only 2 had the MEO with an evaluation score of less than 10, and ~50,4% of the cruises had the MEO with a score of 15 or more. When analysing the combinations of the evaluation scores obtained for the team of MMOs on board (combination of MEO with LEO, see methods), there were 73 unique combinations. From these, only 2 combinations had a cumulative score (MEO + LEO) of less than 10 (Fig. 2).

### Modelling bias on number of sightings recorded per survey

**Fig. 3:**
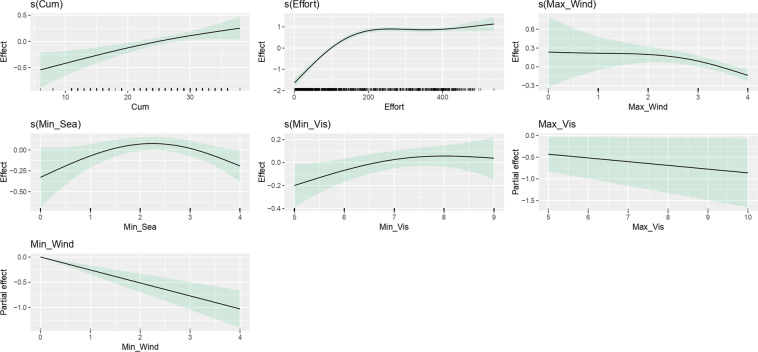
Fitted GAM for number of sightings. Results from the final Generalized Additive Model (GAM) to assess the bias on the number of sightings recorded per survey, fitted with variables related with the survey effort, the meteorological conditions, and the experience of observers. Tick marks above the x-axis indicate the distribution of observations. The area shadowed in green represents the 95% confidence interval of the spline functions. Cum – cumulative evaluation score (scale of 0 to 40); Effort – distance in kilometres sampled on effort per survey; Max_Wind – maximum wind state registered per survey (Beaufort scale); Min_Sea – minimum sea state registered per survey (Douglas scale); Min_Vis – minimum visibility state registered per survey (scale of 0 to 10); Max_Vis – maximum visibility state registered per survey (scale of 0 to 10); Min_Wind – minimum wind state registered per survey (Beaufort scale). For details on variables, see methods.

The final GAM model included the following explanatory variables: kilometres sampled “on-effort”, minimums of the sea state, minimums and maximums of the wind state and visibility, and the cumulative evaluation score of the LEOs and MEOs (see methods for details on the variables; Table 2; Fig. 3).

**Table 2:** Results from the final GAM for number of sightings.

GAM n = 894; Deviance explained = 49.4%; R-square = 0.452; UBRE = 0.008				
Variables	Degrees of Freedom	Reference Degrees of Freedom	Chi-Square	P-value
s(Cum)	1.388	1.662	18.265	<0.001
s(Effort)	2.961	2.999	486.687	<0.001
s(Max_Wind)	1.746	2.069	16.564	<0.001
s(Min_Sea)	2.147	2.538	9.680	0.012
s(Min_Vis)	1.756	2.154	5.637	0.073
**Variables**	**Estimate**	**Standard Error**	**Z-value**	**Pr(**>**|z|)**
Max_Vis	−0.08614	0.04060	−2.122	0.034
Min_Win	−0.25680	0.04678	−5.489	<0.001

Generalized Additive Model (GAM) to assess the bias on the number of sightings recorded per survey, fitted with variables related with the survey effort, the meteorological conditions, and the experience of observers. Cum – cumulative evaluation score (scale of 0 to 40); Effort – distance in kilometres sampled on effort per survey; Max_Wind – maximum wind state registered per survey (Beaufort scale); Min_Sea – minimum sea state registered per survey (Douglas scale); Min_Vis – minimum visibility state registered per survey (scale of 0 to 10); Max_Vis – maximum visibility state registered per survey (scale of 0 to 10); Min_Wind – minimum wind state registered per survey (Beaufort scale). For details on variables, see methods.

The predictors included in the model fitting explain almost half of the number of sightings recorded in a survey (49.4% deviance explained), being the effort the most influent variable (removing effort decreases the deviance explained to 21.1% - see Supplementary Table S2.1).

Overall, number of sightings recorded in a survey increases with the cumulative experience of MMOs and with the effort up to ~200 km (stabilising thereafter). The number peaks with the minimum sea state 2 (in Douglas scale) and generally decreases with higher wind and poorer visibility. The confidence interval for the maximum visibility is very wide (Fig. 3).

### Modelling bias on identification success

**Fig. 4:**
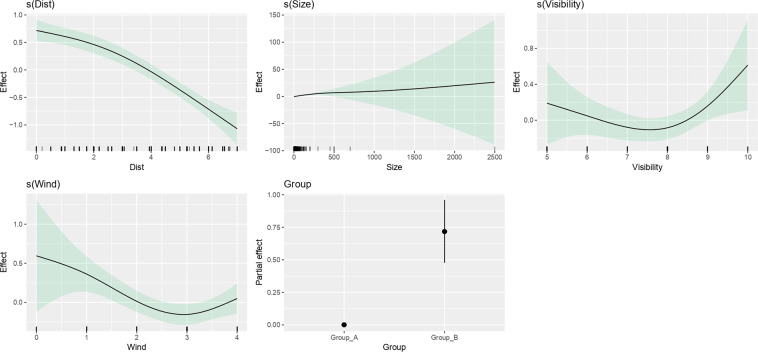
Fitted GAM for identification success. Results from the final Generalized Additive Model (GAM) to assess the bias on the identification success, fitted with variables related with the group size, the distance to the sighting, the meteorological conditions, and the experience of observers. Tick marks above the x-axis indicate the distribution of observations. The area shadowed in green represents the 95% confidence interval of the spline functions. Dist – distance of the sighting to the vessel (scale of 0 to 7); Group – cetacean group of species (Group A – Odontoceti sightings, excluding sperm whales; Group B – Mysticeti sightings, plus sperm whales); Size – group size (i.e., number of animals in the group) of the sighting; Vis – visibility state (scale of 0 to 10). Wind – wind state (Beaufort scale); For details on variables, see methods.

The final GAM model included the following explanatory variables: group of cetaceans (Group A – odontoceti, except sperm whale; Group B – mysticeti, plus sperm whale), group size (number of animals in the group sighted), distance of the sighting to the vessel, wind state, and visibility (see methods for details on the variables; Table 3; Fig. 4).

**Table 3:** Results from the final GAM for identification success.

GAM n = 2496; Deviance explained = 6.06%; R-square = 0.078; UBRE = 0.31				
Variables	Degrees of Freedom	Reference Degrees of Freedom	Chi-Square	P-value
s(Dist)	2.104	2.481	127.372	<0.001
s(Size)	1.238	1.421	63.710	<0.001
s(Vis)	2.340	2.699	9.264	0.048
s(Wind)	2.308	2.646	16.700	0.002
**Variables**	**Estimate**	**Standard Error**	**Z-value**	**Pr(>|z|)**
roup	0.71889	0.12366	5.813	<0.001

Generalized Additive Model (GAM) to assess the bias on the identification success, fitted with variables related with the group size, the distance to the sighting, the meteorological conditions, and the experience of observers. Dist – distance of the sighting to the vessel (scale of 0 to 7); Group – cetacean group of species (Group A – Odontoceti sightings, excluding sperm whales; Group B – Mysticeti sightings, plus sperm whales); Size – group size (i.e., number of animals in the group) of the sighting; Vis – visibility state (scale of 0 to 10). Wind – wind state (Beaufort scale); For details on variables, see methods.

The predictors explained 6.05% of the deviance in the identification success, and the results of the step-by-step backward selection highlighted the relevance of the distance to the sighting, as well as the group size (see Supplementary Table S2.2). Overall, the identification success was higher in Group B and with bigger group sizes (although with a very wide confidence interval for the latter), and decreases with distance to the sighting and with worse meteorological conditions (Fig. 4).

## Discussion

Until now, the monitoring protocol of CETUS did not include the need for photographic/video register of sightings. Priority was given to record position and then to the observation of the animals as much as possible to allow for identification and assessment of the group size. Also, since CETUS is a non-funded project, it is not possible to have cameras with long-zoom lenses on board each ship, especially on a long-term basis. Yet, the photographic/video verification and validation processes here presented proof that these image registers of sightings are fundamental, and their collection needs to be included in the CETUS monitoring protocol. Although we obtained photos from only 315 single-taxon sightings, we were able to match ~91% of these with the dataset. The ~9% we did not match were mainly due to wrong metadata regarding date and time of the photographic/video registers. This issue can easily be solved by correcting the date/time of the on board cameras being used for cetacean monitoring, before each survey. After the photographic validation of the sightings, the updated version of the CETUS dataset has now 223 records validated to the taxon level they are registered with, with 170 of them validated to the species level. The process also allowed to register 49 sightings with a lower taxon than originally recorded by the MMOs, and resulted in the inclusion of a new species to the dataset.

From the completely validated sightings, 10 of them (~6% error rate) had been misidentified by the observers and are now corrected. If we apply the error rate to the entire dataset, we have an additional number of potentially 208 misidentifications that could have been corrected if all sightings were recorded with photos/videos. Ideally, in monitoring programmes based on visual methods, a professional camera should be provided for each platform of observation, but this is not always possible, often due to funding-related issues. However, the promising results on photographic validation, here obtained mostly with poor-quality photos/videos, show that, even registers with non-professional, less expensive, cameras or mobile devices, are useful to substantially reduce the species misidentifications. Therefore, although less-ideal, observation platforms can be equipped with non-professional devices to ensure photographic/video recordings of the sightings. In the case of the CETUS Project, a possible solution is to request MMOs to use the tablet, provided to record the track and data, to collect sighting images. Alternatively, if suitable, the MMOs can be requested to use their personal devices, provided they are then given credit for the images. In this last case, it is crucial that pictures taken on board are collected immediately after the surveys or after the MMOs participation in the monitoring programme. Otherwise, there is a high chance the images are eventually lost, or the contact with observers is not possible in the future. Finally, a small course or guidelines for image recording should always be given to the MMOs, specifically focused on the use of the devices (more so if a professional camera is provided) and how to photograph the animals at sea. The aforementioned procedures (availability of recording devices and course/guidelines) would substantially increase the number of sightings photographed/videotaped, and therefore reducing misidentifications and increasing the records validated. Ultimately, this would improve the dataset quality and the users’ confidence in the data.

Most photographs/videos obtained from MMOs were of delphinid sightings. Delphinids are the most frequently observed cetaceans in the CETUS surveys. They are avid riders of the bow wakes of the ships, some of them performing aerial stunts, such as high leaps and flips, which makes them easier to photograph^19,20^. Most misidentifications (9 out of 10) were among small delphinids, specifically between *Stenella frontalis*, *Stenella attenuata* and *Stenella coeruleoalba*, likely due to their visual similarities^21^. In monitoring programmes, during the training of MMOs, the highly similar species likely to be detected should be flagged, and it should be emphasized to the MMOs the importance of employing a high effort to photograph them at sea.

With the information now available in the 2^nd^ version of the CETUS dataset, related with photographic validation, the users may decide to consider only validated sightings, which may be necessary, for example, when more conservative approaches need to be employed. Since most misidentifications were among the small delphinids of the same genus, the users may also consider using the entire dataset, but merge small delphinid sightings to the genus level.

In the present study, the MMOs’ experience was evaluated and scored based on their *curricula vitae* at the time of the internship, prior to each cruise. In the future, it could be useful to evaluate the MMOs based on their performance in the theoretical trainings, and if possible, in practical trainings at sea. Furthermore, quality criteria could include indexes based on the number of sightings and species recorded by the MMOs throughout their participation in the CETUS campaigns. These indexes should also account for the monitoring time and weather conditions during their period on board. Such methods would probably result in a better proxy of the MMOs’ performance and yield better and more useful evaluation scores. The observers’ fatigue and the survey duration should also be included in future analyses to investigate if there is the need to reduce the monitoring time or, in cases where it is possible, consider a shift-based approach, with more than two MMOs, as often suggested to reduce the fatigue effect on data collection^22^. Overall, the experience of the MMOs in the teams was well-balanced between the most experienced observer compensating and the least experienced observer. With the information of the evaluation scores (of the MMOs’ experience) available in the 2^nd^ version of the CETUS dataset, the users are able to select data based on thresholds to the observers’ experience, if they wish to, for example, use only data collected by more experienced observers.

Bias modelling can provide quantitative information to support the user in selecting the data or defining thresholds, according to their study aims^6^. Here, we assessed bias to the number of sightings collected by the observers per survey, related with sampling effort, detectability factors (i.e., weather), and MMOs’ experience. Sampling effort was the biggest source of bias. In fact, when the study aim is to assess abundance, it is recommended that the survey design ensures a homogeneous coverage of sampling effort in space and time^4,23^. However, this is not always possible, especially when monitoring depends on platforms of opportunity. In these cases, it is crucial that data is standardized according to sampling effort, and it is advised to use measures of encounter rates instead of absolute abundance estimates^24,25^. In the CETUS surveys, the number of sightings collected stabilises after the 200 km of sampled effort. Therefore, users may decide to use data collected only from surveys where over 200 km were sampled. The weather conditions influence detectability, with an overall decreasing in the number of detections with poorer weather conditions (strong wind speed, high waves and poor visibility). An exception is the correlation with sea state, with the number of sightings increasing with wave height, peaking around two (Douglas scale), and decreasing thereafter. Since most sightings in the CETUS dataset belong to the family Delphinidae (~62%), this correlation can be explained by the fact that dolphins tend to wave-ride, and thus may be visible at the surface for longer if the waves are higher and wider. This behaviour is believed to provide additional benefits in terms of speed and energy costs^26^. However, with waves surpassing the 0.50 m height (two in the Douglas scale), the negative effect of sea state on cetacean detectability impedes the observation of animals, even of jumping dolphins. The number of sightings increases with the cumulative evaluation of the MMOs teams (sum of the evaluation scores of the MMOs). To combine a less experienced observer with a very experienced one is a good strategy to provide quality training to the least experienced observer, without substantially losing data quality. Overall, the explanatory variables included in the model explained a great amount of deviance in the number of sightings (~49%). This highlights the importance to apply bias modelling in data obtained from visual methods. The results here presented provide additional information to the CETUS dataset, allowing users to select the data depending on the needs of the analysis intended: i.e., if a very conservative approach is deemed necessary, a user may choose to select data from surveys with over 200 km, sea state and wind state below 2 (Douglas and Beaufort scale, respectively), visibility higher than 4000 m, and a score of the MMOs cumulative evaluation higher than 20.

The bias to the success in species identification were assessed in relation to the characteristic of the sightings (distance of the sighting, species/group type, group size) and to the detectability factors (i.e., weather). The modelling results show that the distance of the sighting to the vessel is the variable that mostly affects identification ability. As expected, the success in the species identification when animals are closer to the boat, and decreases with worse weather conditions (wind and visibility). Successful identifications are related with size of animals and groups (higher success with baleen and sperm whales, and with bigger groups of animals). The use of binoculars is essential to support the identification of the species, especially with sightings at a distance. With long hours of observation effort, it is easy to forget or underrate the use of binoculars, especially when the movement of the vessel and weather conditions interfere with their stabilisation. Therefore, it is worth emphasizing to the observers the importance of using binoculars during visual monitoring to increase identification success. Bias modelling of the identification success only explained ~6% of the variance with the selected explanatory variables. It is likely that the success rate is mostly explained by random factors, not possible to determine *a posteriori*. Additional data that could be collected upon monitoring to test their impact on identification success is: behaviour of the animals (beneath surface, aerial, surfing), individual characteristics (if the animal presents the most identifiable characteristics of the species, such as coloration patterns, size or shape), or duration of the sighting. Other factors, less obvious and more difficult to register during monitoring are, for example, the predisposition of the observer to the surveying work. A random weight can also be attributed to the data when performing statistical analyses such as ecological modelling, to account for any of these random factors not collected during surveys^27^. Although with a low deviance explained, these modelling results can support the usage of the data. The user may decide to select data only collected under optimal conditions, for example, selecting data records according to a threshold in the sighting distance, as this was the strongest bias to the identification success.

This work highlights the relevance of applying verification methods to datasets, and to assess and quantify data bias. In terms of applicability, it made available valuable information to allow for an informed usage of the CETUS dataset, and guidelines to improve data collection and processing of programmes based on visual methods for species monitoring.

## Methods

### Data processing

In 2019, the CETUS data spanning between 2012 and 2017 was published open access through the Flanders Marine Institute (VLIZ) IPT portal and distributed by EMODnet and OBIS, in a first version of the CETUS dataset^9^. The data collected between 2018 and 2019 was prepared as the 2012–2017 data^9^. Methods for photographic verification/validation and to evaluate the MMOs experience were applied (see below), in order to include new variables on data quality in an updated version of the dataset. Currently, the CETUS dataset is updated, with a 2^nd^ version available^10^. It comprises data from 2012 to 2017, with the following two new columns on the observers’ experience: “most experienced observer” and “least experienced observer”; and a new column associated with validation of the sightings’ identifications: “photographic validation”. The results here presented correspond to the analysis of the data from 2012 to 2019, and the open-access dataset will soon be further updated with the 2018–2019 data.

### Photographic verification

All the former MMOs who have integrated the CETUS Project, between 2012 and 2019, were contacted and asked to provide any available photographic or video records of cetaceans collected during their on board periods. The collection of sighting’s images was not a requirement of the CETUS protocol, and so these records were obtained opportunistically, with availability and quality depending on several factors: observers on board having personal cameras, camera quality, intention of the observer taking the photograph (e.g., for aesthetic or identification purposes).

The images obtained were organized in a folder hierarchy from the year to the day of recording. However, not all the images had metadata up to the day of recording, so these were inserted into the most appropriate hierarchy-level of the folder organization. For each set of records corresponding to a single-taxon sighting, the photos/videos with the better quality or framing (i.e., that allowed for an easier species identification) were selected for that sighting. The remaining photos/videos were only consulted in case of doubt (e.g., to look for additional details that could help with the identification).

Verification consisted of the process of matching the photographic/video records with the dataset sighting registers. Whenever possible and ideally, the file metadata was used for the process. However, often, the date and/or time of the file metadata were wrong, non-existent, or in different time zones. In these cases, a conservative methodology was applied using all available information to match as many sightings as possible. An estimation of time lag was attempted (based on, at least, two obvious matches between photographs/videos and dataset registers, e.g., unique sighting of the day, close to the boat, easy/obvious identification). When not possible, further evaluation consisted in assessing whether the sighting and image record was too obvious, and accounting for unique complementary information on the sighting (e.g., the number of animals or the side of the sighting were unique for that day and/or for that species/group).

### Photographic validation

After the verification process, the validation of the matched records was carried out, to confirm or correct the species identification of sightings in the 1^st^ version of the CETUS dataset (i.e., reported by the MMOs on board). The validation approach involved, for more dubious identification through the photo/video records, the discussion between four experienced observers of the CETUS team. In cases where no consensual agreement was achieved, an external expert on cetacean identification was also consulted. Identifications made through the photographic/video records required 100% certainty, and these were then compared with the cetacean identifications provided in the 1^st^ version of the CETUS dataset. Then, the occurrence records with originally misidentifications of cetaceans, as well as those records where validation allowed to achieve an identification to a lower taxon, were corrected in the 2^nd^ version of the dataset (i.e., a delphinid sighting validated as common dolphin, will now appear as common dolphin). A new column “photographic validation” was added to the dataset with the following categories: “yes” (i.e., validated with photograph/video), “no” (i.e., not validated with photograph/video), and “to the family” (i.e., validation only to the family taxon).

For further analysis, specifically for the model process on the identification success (see below), registers were considered “completely validated” if it was possible to complete the photographic/video identification process up to the species level (then, differentiating if the original identification from the MMOs was or not correct). For *Globicephala* sp. and *Kogia* sp., validation to the genus was considered complete, since the species from both genera are visually hardly differentiated, especially at sea.

### Creating a data quality criteria: evaluating MMOs experience

Quality criteria were created to evaluate the MMOs experience based on the information collected from their *curricula vitae* (CVs) (alumni MMOs provided as many CVs as the years of their participation in CETUS). The following quality criteria were considered: (i) the experience at sea, (ii) the experience with cetaceans’ ID, (iii) the number of species they have worked with, and (iv) the experience working with the CETUS Project protocol. Each of these quality criteria was ranked from 0 to 5, and then these were summed to generate an evaluation score, on a scale of 0 to 20, attributed to each MMO (Table 4).

**Table 4:** Quality criteria for MMOs evaluation.

Experience at sea		Experience with CETUS Project protocol	
Owns diving/lifeguard courses	1	Up to 1 month on board	1
Has up to 3 months of experience on small boats	2	More than 1 month and up to 3 months on board	2
Has more than 3 months of experience on small boats	3	More than 3 months and up to 5 months on board	3
Has up to 3 months of experience on ships	4	More than 5 months and up to 7 months on board	4
Has more than 3 months of experience on ships	5	More than 7 months on board	5
**Number of species MMOs have worked**		**Experience with cetaceans’ ID**	
Research (desk-based, lab or fieldwork) directed to 1 species only with apparently no contact with other species	1	Up to 3 months of experience with cetaceans (e.g., whale watching, cetaceans monitoring or cetacean’s rehabilitation)	1
Research (desk-based, lab or fieldwork) directed to 1 or 2 species in areas with a medium number of species (e.g., Mediterranean)	2	More than 3 months and up to 6 months of experience with cetaceans	2
Research (desk-based, lab or fieldwork) directed at 1 or 2 species in areas with a high number of species (e.g., Canaries or Azores)	3	More than 6 months and up to 1 year of experience with cetaceans	3
Generalized research (desk-based, lab or fieldwork) of species in areas with a medium number of species	4	More than 1 year and up to 3 years of experience with cetaceans	4
Generalized research (desk-based, lab or fieldwork) of species in areas with a high number of species	5	More than 3 years of experience with cetaceans	5

Quality criteria created to evaluate the experience of the CETUS Project Marine Mammal Observers (MMOs) and to generate an evaluation score for the least and most experienced observer on board (generally, two MMOs on board). ID – Identification.

The MMOs evaluations were computed for each cruise (i.e., the trip from one port to another), considering the experience of the MMOs based on the CV obtained for that year, plus the experience acquired during CETUS participation in previous cruises that year. Since most of the times, the team of observers on board each cruise was constituted by two MMOs, two final evaluation scores were attributed to each cruise in the 2^nd^ version of the CETUS dataset, into two new columns: “most experienced observer” and “least experienced observer”. On rare occasions where there is only one observer on board that cruise, only the evaluation of the single observer was included under the column “most experienced observer”, leaving the column “least experienced observer” as “NULL”. To investigate the experience of MMOs on board, both individually and cumulative (LEO + MEO), the combination of the score values was computed by cruise. These were then trimmed to unique combinations of evaluation scores.

The names of observers, previously presented in the online dataset for each cruise, were removed for anonymity purposes, as there is now ancillary information regarding their experience.

### Model fitting

Two Generalized Additive Models (GAM) were fitted to assess bias on the number of sightings recorded per survey and on the identification success of cetacean species. Details for each model are presented below. Both models were fitted in R (Version 4.1.0). Prior to modelling, Pearson correlations were calculated between all pairs of explanatory variables, considered for each model (see below), to exclude highly correlated variables, considering a threshold of 0.75^24,25,28^. Since the variables regarding MMOs’ experience were correlated (LEO or MEO correlated with cumulative and mean experience; and cumulative experience correlated with mean experience – Supplementary Fig. S3), these variables were not included in the first fitting stage (backward selection) but included later through forward selection (see below). Multicollinearity among explanatory variables was measured through the Variance Inflation Factor (VIF), with a threshold of 3 (Supplementary Tables S4)^24,25,29^. After removing the MMOs evaluation scores, no multicollinearity was observed, so all the other variables were kept for the first fitting stage.

For model selection, a backward selection was applied to oversaturated models^18,24,25,30,31^. The Akaike Information Criterion (AIC) was used as a measure of adequation of fitness, choosing the model with the lowest AIC value at each step of the model fitting process, i.e., comparing nested models (larger model incorporating one more explanatory variable compared with the smaller model). If the AIC-difference between the two models was less than 2, an Analysis of Variance (ANOVA), through chi-square test, was used to check if the AIC-difference was significant^24,25,32^. If this difference was not statistically significant (p > 0.05), the simplest model (smaller model) was kept. Through a forward selection process, the variables regarding the MMOs evaluation scores were added, one at a time, to the best model obtained in the previous backward selection. After comparing the models with each other (separate variables for LEO + MEO vs. Cumulative Evaluation vs Mean Evaluation), the best model, considering the AIC value, was kept. A final backward selection process was then applied.

All GAMs were fitted with the “mgcv” package (https://cran.r-project.org/web/packages/mgcv) and a maximum of four splines (k = 4) was chosen to limit the complexity of smoothers describing the effects of the explanatory variables^25,31^. If a spline was close to linear (with estimated degrees of freedom of ~1), the smooth term was removed, and a linear function was fitted. To check for model quality, the “gam.check” function was used to verify the diagnostic plots and the adequacy of the number of splines (Supplementary Figs. S5 and S6). Existence of influential data points was assessed (with the threshold of 0.25 for the Hat values), as well as the correlation between model residuals and explanatory variables. In both final models, number of splines was adequate and there were no influential data points or clear correlation between residuals and explanatory variables (Supplementary Figs. S7 and S8)^24,32^.

#### Bias modelling of number of sightings

To assess the bias parameters on the number of sightings recorded per survey (i.e., a full day monitoring, from sunrise to sunset), the following detectability factors were considered as explanatory variables: weather conditions (i.e., the minimums and maximums of the sea state, wind state, and visibility), the experience of MMOs (i.e., the evaluation scores of the least and the most experienced observers, as well as the mean and cumulative evaluations of the MMOs experience) and kilometres sampled “on-effort” (i.e., periods of active survey). Sampling periods were divided into “On-effort” and “Off-effort” conditions, based on four meteorological variables: sea state (Douglas scale), wind state (Beaufort scale), visibility (measured in a categorical scale ranging from 0–10 and estimated from the distance to the horizon line and possible reference points at a known range, e.g., ships with an automatic identification system, > 1000 km), and the occurrence of rain (see Supplementary Table S9)^10^. For the model fitting, only “on-effort” periods of sampling were considered. Given that the response variable was count data, a Poisson distribution was tested (with a log link function). Then, the resulting first oversaturated model was checked for overdispersion, through a Pearson estimator. Since it tested positive for overdispersion (φ = 1.99), a negative binomial distribution (with a log link function) was fitted.

#### Bias modelling of identification success

A binary response variable, based on the success in the species identification for each sighting, was generated, and a model with binomial distribution (with a logit link function) was fitted. As in the previous model, only “on-effort” records were used. The total number of non-successful identifications across the dataset (the 0 s of the model) was extrapolated from the proportion of wrong identifications obtained in the validation process. To calculate this proportion, only complete validated sightings registered “on-effort” were used. Proportions were computed and extrapolated to Odontoceti and Mysticeti, separately. This resulted in 78 non-successful identifications in delphinids, plus 17 misidentifications in baleen whales, i.e., a total of 95 “on-effort” sightings randomly selected from the dataset were defined as unsuccessful identifications (0 s in the response variable for model fitting). The remaining records were considered successful identifications (1 s in the response variable for model fitting). To assess the bias parameters on the identification success, the following independent variables were considered in the analysis: the group (i.e., Group A: Odontoceti sightings, excluding sperm whale (*Physeter macrocephalus*); and Group B: Mysticeti sightings, plus sperm whale), the size of the group (i.e., the best estimate of the number of animals in a sighting, from the observer’s perspective), sighting distance (i.e., a relative measure according to the scale of the binoculars), weather conditions (i.e., the sea state, wind state, and visibility at the time of each sighting), the experience of MMOs (i.e., the evaluation scores of least and most experienced observers, as well as the mean and cumulative scores of the MMOs teams). Group A and B were settled according to cetacean morphology. However, since sperm whales have closer similarities with Mysticeti species, they were also included in Group B^21,33^. This categorization was mostly based on body size, as this is likely the main factor, regarding the species morphology, influencing the identification. Group A is constituted by species with a medium length of less than 10 meters, while Group B includes the larger species over 10 meters (Mysticeti plus *P. macrocephalus*)^33^. Since in the CETUS Project, different binoculars have been used - with two different reticle scales - it was necessary to standardize binocular distances to the same scale.

## Supplementary information

Supplementary Information

## Data Availability

The data used to support the findings is partially (2012–2017) available at 10.14284/547^10^ and can be downloaded as a self-contained file (Darwin Core Archive, DwC-A)^10^. As described in the CETUS dataset data descriptor, this includes information about the sampling events (i.e., geographic coordinates, date, protocol, etc.), the occurrences (taxonomical information) and the biological/environmental measurements. These are stored in three separate files: i) Events, ii) Occurrence and iii) ExtendedMeasurementOrFact^9^. The data collected between 2018 and 2019 will be published in the next update of the CETUS dataset.

## Code availability

The code scripts used to build the models under R software are available at the supplementary material (see Supplementary Information S102) and open-source, archived at GitHub, with Zenodo, at: 10.5281/zenodo.7139034^34^.
