# Supplementary material for: Assessing data bias in visual surveys from a cetacean monitoring programme
Source: Sci Data. 2022 Nov 10;9:682. doi: 10.1038/s41597-022-01803-7 (PMC9649672; doi:10.1038/s41597-022-01803-7)
Supplement: Supplementary file 1 — Supplementary Information [file 41597_2022_1803_MOESM1_ESM.pdf]

Supplementary Information

Table of Contents

Supplementary Table S1. Updated number of cetacean occurrences.. 2
Supplementary Tables S2. Backward selection of the Generalized Additive Models (GAMs)...... 3
Supplementary Figures S3. Correlation Matrices. 5
Supplementary Tables S4. Variance Inflation Factor (VIF) results. 7
Supplementary Figures S5. Diagnostic plots (gam.check). 8
Supplementary Tables S6. Results of basis dimension (k) (gam.check)....................................... 9
Supplementary Figures S7. Influence plots.. 10
Supplementary Figures S8. Residual plots. 11
Supplementary Table S9. Meteorologic variables assessed during CETUS Project surveys. 13
Supplementary Information S10. Code scripts. 14

Supplementary Table S1. Updated number of cetacean occurrences.

These are the single-taxon sightings, recorded between 2012 and 2019, of the CETUS dataset after the video / photographic verification and validation processes. The Taxon Rank is in agreement with the World Register of Marine Species (WoRMS, https:// https://www.marinespecies.org/). Within brackets is the difference in number of occurrences of the updated CETUS dataset in relation to the version before the photographic / video verification and validation. For the validated records, the number of complete validations is presented and, within brackets, the number of partial validation ("to the family"). The table is organized by taxon rank of the occurrences (recorded to the lowest taxon level), and alphabetically within.

Taxa	Taxon Rank	No of Occurrences	No of validated
Cetacea	Infraorder	425 (-5)	3
Balaenopteridae	Family	356 (-6)	1
Delphinidae	Family	1002 (-20)	30
Ziphiidae	Family	177 (-5)	15
Balaenoptera	Genus	5 (+5)	5
Globicephala	Genus	70 (+1)	15 (1)
Kogia	Genus	8 (+1)	1
Balaenoptera acutorostrata	Species	94	1
Balaenoptera borealis	Species	5 (+1)	1
Balaenoptera edeni	Species	6	0
Balaenoptera musculus	Species	3	0 (1)
Balaenoptera physalus	Species	38 (+2)	2
Delphinus delphis	Species	481 (+5)	25 (5)
Grampus griseus	Species	13 (+1)	0
Hyperoodon ampullatus	Species	6	0 (2)
Lagenodelphis hosei	Species	1	0
Lagenorhynchus albirostris	Species	3	0
Megaptera novaeangliae	Species	9	0
Mesoplodon densirostris	Species	8	0 (1)
Mesoplodon europaeus	Species	4 (+4)	4
Orcinus orca	Species	8	2
Peponocephala electra	Species	4	1
Phocoena phocoena	Species	9	0
Physeter macrocephalus	Species	179 (-1)	16
Pseudorca crassidens	Species	13	0 (2)
Stenella attenuata	Species	7 (-2)	0 (1)
Stenella clymene	Species	17	0
Stenella coeruleoalba	Species	185 (+4)	25
Stenella frontalis	Species	320 (+13)	54 (8)
Stenella longirostris	Species	6	0
Steno bredanensis	Species	4	1
Tursiops truncatus	Species	172 (-1)	14 (1)
Ziphius cavirostris	Species	82 (+3)	7 (5)
**Total**	**33 taxa**	**3720 occurrences**	**223 (+27) validated**

Supplementary Tables S2. Backward selection of the Generalized Additive Models (GAMs).

Supplementary Table S2.1. Backward selection of the Generalized Additive Model (GAM) to assess the bias on the number of sightings recorded per survey, fitted with variables related with the survey effort, the meteorological conditions, and the experience of observers. Cum – cumulative evaluation score (scale of 0 to 40); Effort – distance in kilometres sampled on effort per survey; Max_Vis – maximum visibility state registered per survey (scale of 0 to 10); Max_Sea – maximum sea state registered per survey (Douglas scale); Max_Wind – maximum wind state registered per survey (Beaufort scale); Min_Vis – minimum visibility state registered per survey (scale of 0 to 10); Min_Sea – minimum sea state registered per survey (Douglas scale); Min_Wind – minimum wind state registered per survey (Beaufort scale); R-sq – coefficient of determination; UBRE – Un-Biased Risk Estimator; AIC – Akaike Information Criterion. For details on variables, see methods.

**Backward Selection**				
Variables	R-sq	UBRE	Deviance Explained	AIC
Cum; Effort; Max_Wind; Min_Sea; Max_Sea; Min_Vis; Max_Vis; Min_Wind (n = 894)	0.452	0.085434	49.4%	3394.607
**Cum; Effort; Max_Wind; Min_Sea; Min_Vis Max_Vis; Min_Wind** (n = 894)	**0.452**	**0.083204**	**49.4%**	**3392.614**
Cum; Effort; Max_Wind; Min_Sea; Max_Vis; Min_Wind (n = 894)	0.454	0.086405	49.1%	3395.475
Cum; Effort; Max_Wind; Min_Sea; Min_Vis; Min_Wind (n = 894)	0.455	0.08555	49.2%	3394.710
Cum; Effort; Max_Wind; Min_Vis; Max_Vis; Min_Wind (n = 894)	0.444	0.091424	48.8%	3399.962
Effort; Max_Wind; Min_Sea; Min_Vis; Max_Vis; Min_Wind (n = 894)	0.441	0.098596	48.5%	3406.374
Cum; Effort; Min_Sea; Min_Vis; Max_Vis; Min_Wind; (n = 894)	0.429	0.099178	48.5%	3406.894
Cum; Effort; Max_Wind; Min_Sea; Min_Vis; Max_Vis (n = 894)	0.424	0.11504	47.7%	3421.074
Cum; Max_Wind; Min_Sea; Min_Vis; Max_Vis; Min_Wind; (n = 894)	0.2	0.67498	21.1%	3921.658

Supplementary Table S2.2. Backward selection of the Generalized Additive Model (GAM) to assess the bias on the identification success, fitted with variables related with the group size, the distance to the sighting, the meteorological conditions, and the experience of observers. Cum – cumulative evaluation score (scale of 0 to 40); Dist – distance of the sighting to the vessel (scale of 0 to 7); Group – cetacean group of species (Group A – Odontoceti sightings, excluding sperm whales; Group B – Mysticeti sightings, plus sperm whales); Size – group size (i.e., number of animals in the group) of the sighting; Vis – visibility state (scale of 0 to 10). Wind – wind state (Beaufort scale); R-sq – coefficient of determination; UBRE – Un-Biased Risk Estimator; AIC – Akaike Information Criterion. For details on variables, see methods.

**Backward Selection**				
Variables	R-sq	UBRE	Devianced Explained	AIC
Group; Size; Dist; Sea; Wind; Vis; Cum (n = 2396)	0.0775	0.31092	6.11%	3140.972
Group; Size; Dist; Sea; Wind; Vis (n = 2396)	0.0776	0.31045	6.08%	3139.845
**Group; Size; Dist; Wind; Vis** (n = 2396)	**0.0777**	**0.30993**	**6.06%**	**3138.590**
Group; Size; Dist; Wind (n = 2396)	0.0749	0.31192	5.77%	3143.35
Group; Size; Dist; Vis (n = 2399)	0.0719	0.31503	5.56%	3154.76
Size; Dist; Wind; Vis (n= 2673)	0.0997	0.27111	7.78%	3397.684
Group; Size; Wind; Vis (n = 2400)	0.023	0.36598	1.89%	3278.348
Group; Dist; Wind; Vis (n = 2396)	0.0449	0.34367	3.53%	3219.426

Supplementary Figures S3. Correlation Matrices.

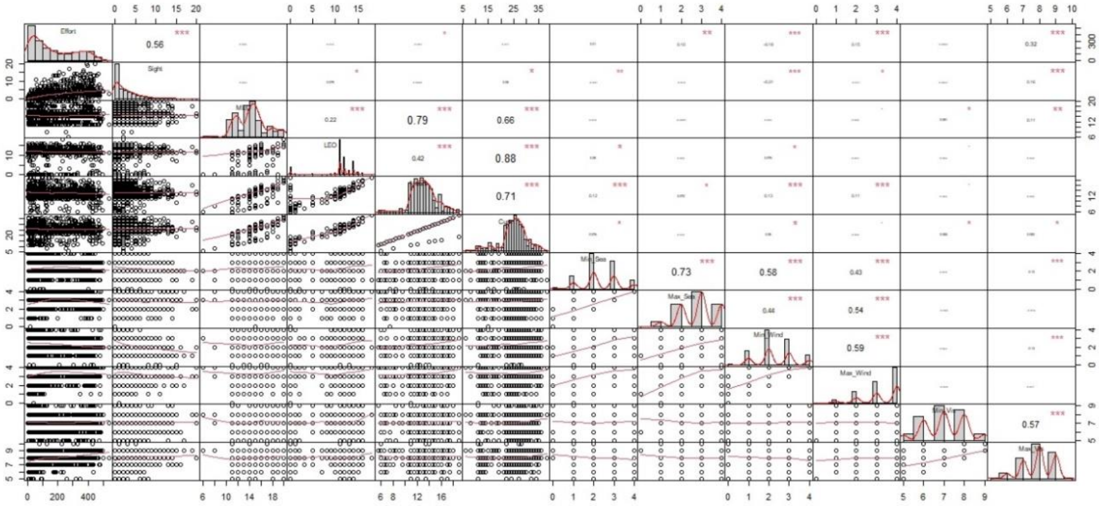

Supplementary Figure S3.1. Results of the pairwise Pearson correlations between explanatory variables for the Generalized Additive Model (GAM) to assess the bias on the number of sightings recorded per survey, fitted with variables related with the survey effort, the meteorological conditions, and the experience of observers. Cum – cumulative evaluation score (scale of 0 to 40); Effort – distance in kilometres sampled on effort per survey; MEO – evaluation score of the Most Experienced Observer per survey (scale of 0 to 20); LEO – evaluation score of the Least Experienced Observer per survey (scale of 0 to 20); Mean – mean of the evaluation scores (scale of 0 to 20); Max_Vis – maximum visibility state registered per survey (scale of 0 to 10); Max_Sea – maximum sea state registered per survey (Douglas scale); Max_Wind – maximum wind state registered per survey (Beaufort scale); Min_Vis – minimum visibility state registered per survey (scale of 0 to 10); Min_Sea – minimum sea state registered per survey (Douglas scale);; Min_Wind – minimum wind state registered per survey (Beaufort scale); R-sq – coefficient of determination; UBRE – Un-Biased Risk Estimator; AIC – Akaike Information Criterion. For details on variables, see methods.

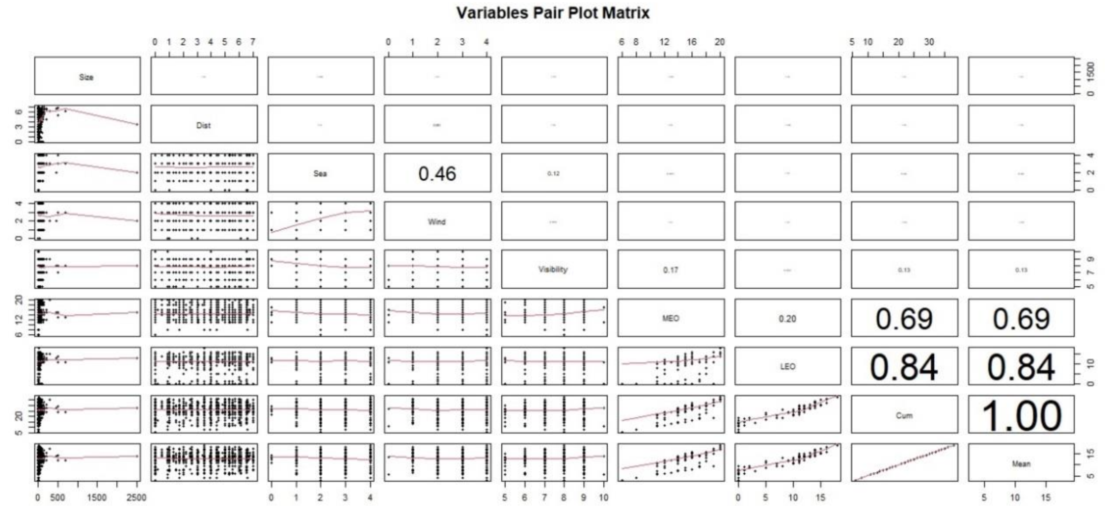

Supplementary Figure S3.2. Results of the pairwise Pearson correlations between explanatory variables for the Generalized Additive Model (GAM) to assess the bias on the identification success, fitted with variables related with the group size, the distance to the sighting, the meteorological conditions, and the experience of observers. Cum – cumulative evaluation score (scale of 0 to 40); Dist – distance of the sighting to the vessel (scale of 0 to 7); Group – cetacean group of species (Group A – Odontoceti sightings, excluding sperm whales; Group B – Mysticeti sightings, plus sperm whales); Size – group size (i.e., number of animals in the group) of the sighting; Vis – visibility state (scale of 0 to 10). Wind – wind state (Beaufort scale); R-sq – coefficient of determination; UBRE – Un-Biased Risk Estimator; AIC – Akaike Information Criterion. For details on variables, see methods.

Supplementary Tables S4. Variance Inflation Factor (VIF) results.

Supplementary Table S4.1. VIF results to test multicollinearity of the explanatory variables for the Generalized Additive Model (GAM) to assess the bias on the number of sightings recorded per survey, fitted with variables related with the survey effort, the meteorological conditions, and the experience of observers. Effort – distance in kilometres sampled on effort per survey; Max_Vis – maximum visibility state registered per survey (scale of 0 to 10); Max_Sea – maximum sea state registered per survey (Douglas scale); Max_Wind – maximum wind state registered per survey (Beaufort scale); Min_Vis – minimum visibility state registered per survey (scale of 0 to 10); Min_Sea – minimum sea state registered per survey (Douglas scale); Min_Wind – minimum wind state registered per survey (Beaufort scale); R-sq – coefficient of determination; UBRE – Un-Biased Risk Estimator; AIC – Akaike Information Criterion. For details on variables, see methods.

VIF Test Results						
**Effort**	**Min_Sea**	**Max_Sea**	**Min_Wind**	**Max_Wind**	**Min_Vis**	**Max_Vis**
1.321457	2.778358	2.593228	2.225509	2.003804	1.672010	1.880839

Supplementary Table S4.2. VIF results to test multicollinearity of the explanatory variables for the Generalized Additive Model (GAM) to assess the bias on the identification success, fitted with variables related with the group size, the distance to the sighting, the meteorological conditions, and the experience of observers. Dist – distance of the sighting to the vessel (scale of 0 to 7); Size – group size (i.e., number of animals in the group) of the sighting; Vis – visibility state (scale of 0 to 10). Wind – wind state (Beaufort scale); R-sq – coefficient of determination; UBRE – Un-Biased Risk Estimator; AIC – Akaike Information Criterion. For details on variables, see methods.

VIF Test Results				
**Size**	**Dist**	**Sea**	**Wind**	**Vis**
1.001977	1.011471	1.281592	1.276097	1.015935

Supplementary Figures S5. Diagnostic plots (gam.check).

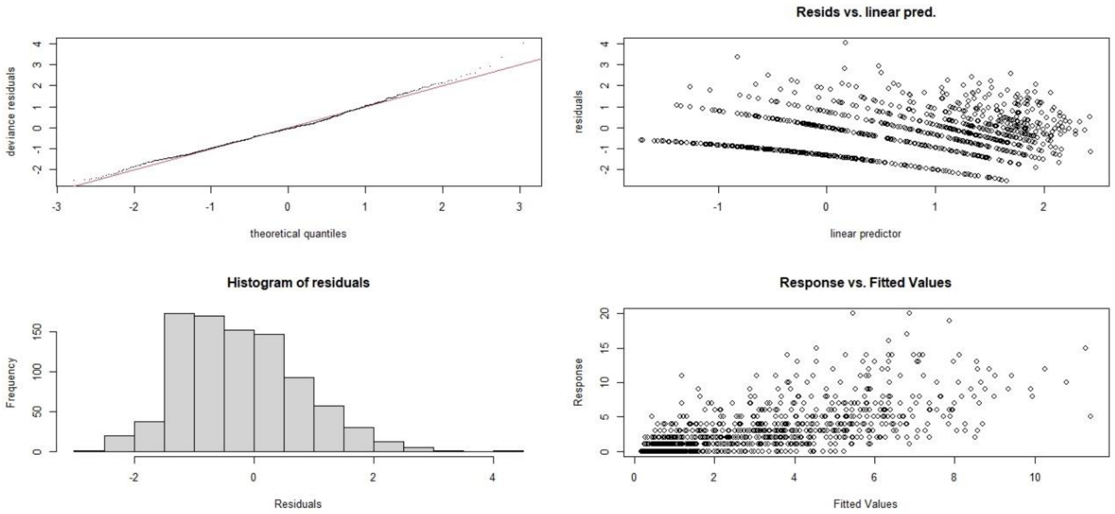

Supplementary Figure S5.1. Diagnostic plots of the final best Generalized Additive Model (GAM) to assess the bias on the number of sightings recorded per survey, fitted with variables related with the survey effort, the meteorological conditions, and the experience of observers.

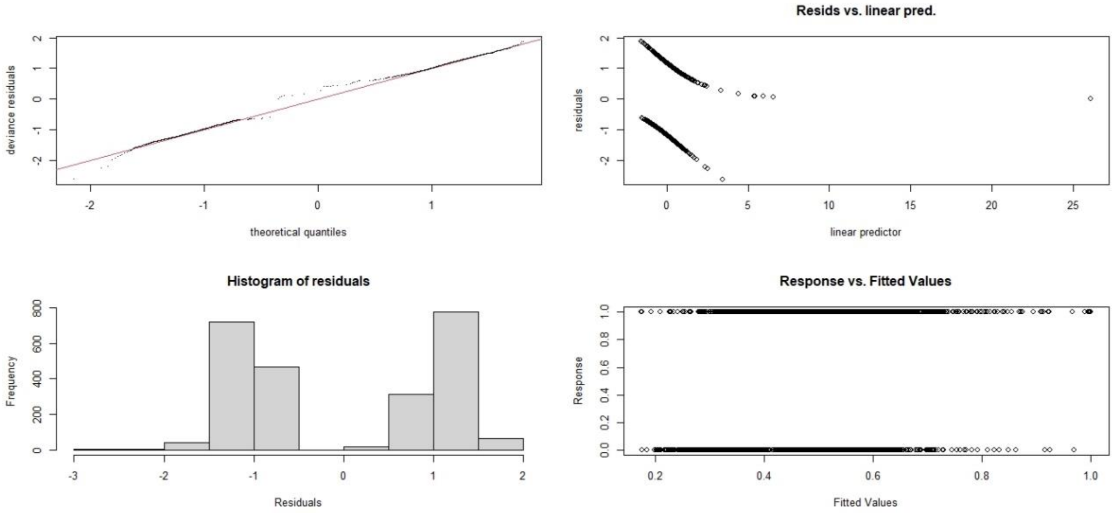

Supplementary Figure S5.2. Diagnostic plots of the final best Generalized Additive Model (GAM) to assess the bias on the identification success, fitted with variables related with the group size, the distance to the sighting, the meteorological conditions, and the experience of observers.

Supplementary Tables S6. Results of basis dimension (k) (gam.check).

Supplementary Table S6.1. Results of basis dimension of the final best Generalized Additive Model (GAM) to assess the bias on the number of sightings recorded per survey, fitted with variables related with the survey effort, the meteorological conditions, and the experience of observers. EDF – Degrees of Freedom; Cum – cumulative evaluation score (scale of 0 to 40); Effort – distance in kilometres sampled on effort per survey; Max_Wind – maximum wind state registered per survey (Beaufort scale); Min_Vis – minimum visibility state registered per survey (scale of 0 to 10); Min_Sea – minimum sea state registered per survey (Douglas scale). For details on variables, see methods.

GAM Check				
**Variables**	**K'**	**EDF**	**K-index**	**P-value**
s(Effort)	3.00	2.96	0.92	0.155
s(Min_Sea)	3.00	2.15	0.88	0.030
s(Max_Wind)	3.00	1.75	0.86	0.005
s(Min_Vis)	3.00	1.76	0.84	$<2x10^{-16}$
s(Cum)	3.00	1.39	0.85	$<2x10^{-16}$

Supplementary Table S6.2. Results of basis dimension of the final best Generalized Additive Model (GAM) to assess the bias on the identification success, fitted with variables related with the group size, the distance to the sighting, the meteorological conditions, and the experience of observers. EDF – Degrees of Freedom; Dist – distance of the sighting to the vessel (scale of 0 to 7); Size – group size (i.e., number of animals in the group) of the sighting; Vis – visibility state (scale of 0 to 10). Wind – wind state (Beaufort scale). For details on variables, see methods.

GAM Check				
**Variables**	**K'**	**EDF**	**K-index**	**P-value**
s(Size)	3.00	1.24	0.09	$<2x10^{-16}$
s(Dist)	3.00	2.10	0.08	$<2x10^{-16}$
s(Wind)	3.00	2.31	0.04	$<2x10^{-16}$
s(Vis)	3.00	2.34	0.05	$<2x10^{-16}$

Supplementary Figures S7. Influence plots.

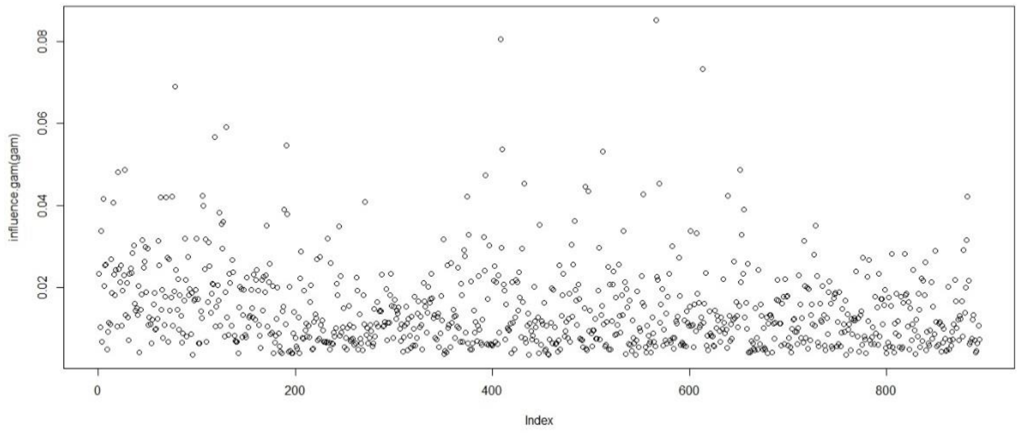

Supplementary Figure S7.1. Influence plot of the final best Generalized Additive Model (GAM) to assess the bias on the number of sightings recorded per survey, fitted with variables related with the survey effort, the meteorological conditions, and the experience of observers.

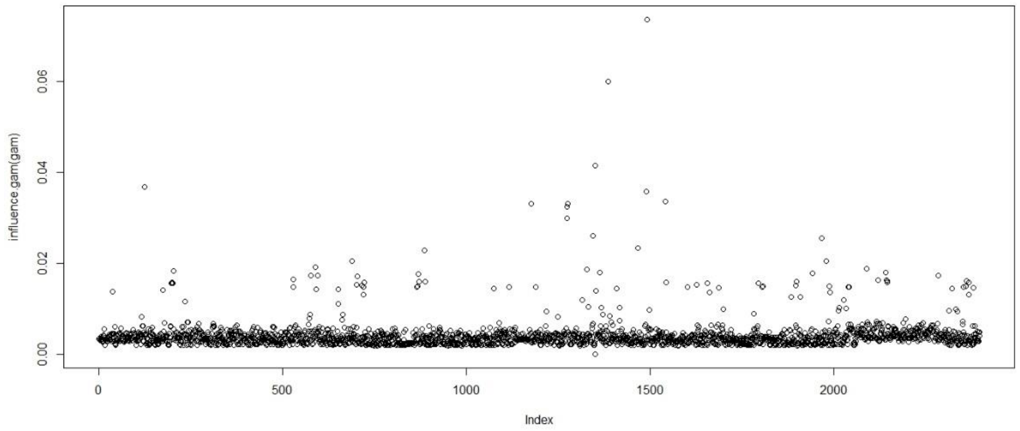

Supplementary Figure S7.2. Influence plot of the final best Generalized Additive Model (GAM) to assess the bias on the identification success, fitted with variables related with the group size, the distance to the sighting, the meteorological conditions, and the experience of observers.

Supplementary Figures S8. Residual plots.

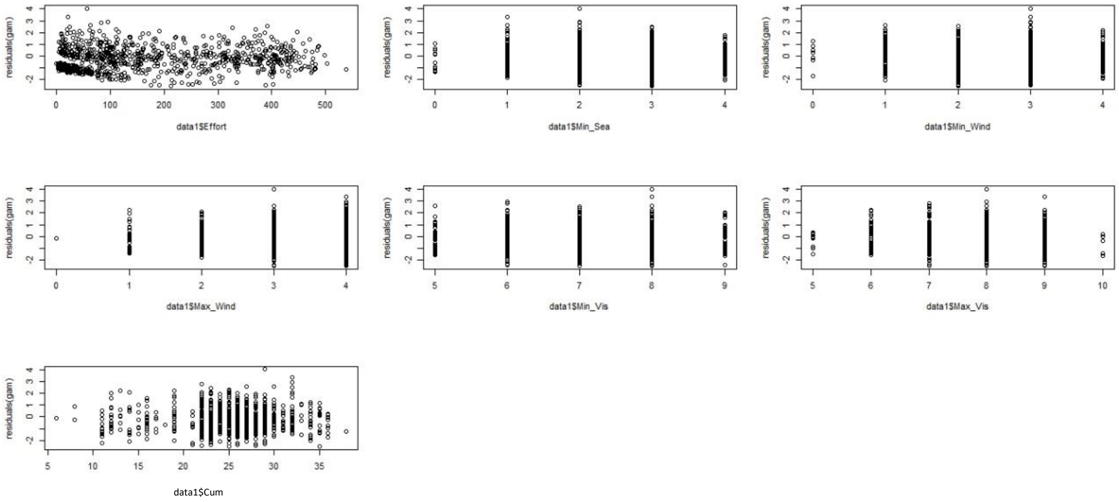

Supplementary Figure S8.1. Residual plots of the final best Generalized Additive Model (GAM) to assess the bias on the number of sightings recorded per survey, fitted with variables related with the survey effort, the meteorological conditions, and the experience of observers. Cum – cumulative evaluation score (scale of 0 to 40); Effort – distance in kilometres sampled on effort per survey; Max_Vis – maximum visibility state registered per survey (scale of 0 to 10); Max_Wind – maximum wind state registered per survey (Beaufort scale); Min_Vis – minimum visibility state registered per survey (scale of 0 to 10); Min_Sea – minimum sea state registered per survey (Douglas scale). For details on variables, see methods.

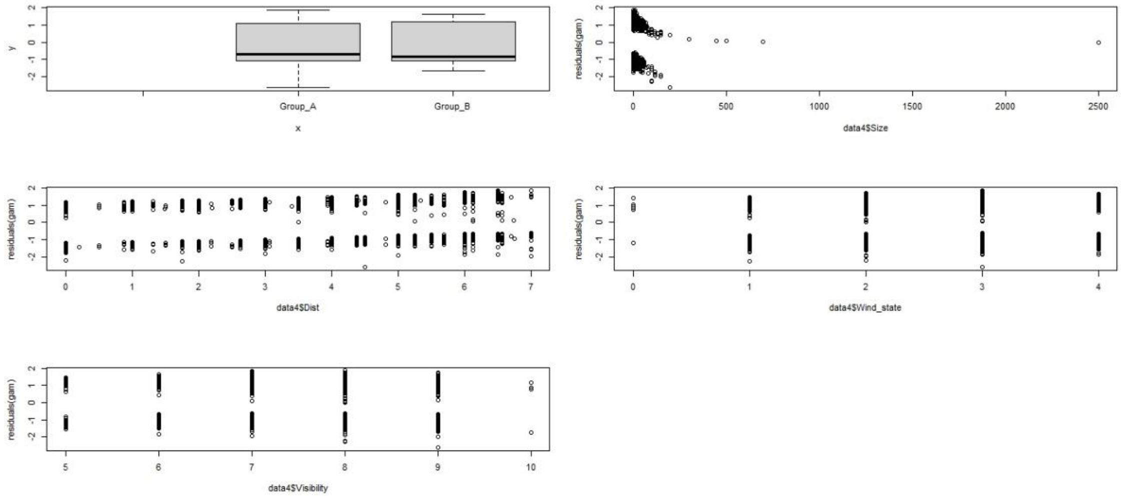

Supplementary Figure S8.2. Residual plots of the final best Generalized Additive Model (GAM) to assess the bias on the identification success, fitted with variables related with the group size, the distance to the sighting, the meteorological conditions, and the experience of observers. Dist – distance of the sighting to the vessel (scale of 0 to 7); Group – cetacean group of species (Group A – Odontoceti sightings, excluding sperm whales; Group B – Mysticeti sightings, plus sperm whales); Size – group size (i.e., number of animals in the group) of the sighting; Vis – visibility state (scale of 0 to 10). Wind – wind state (Beaufort scale).

Supplementary Table S9. Meteorologic variables assessed during CETUS Project surveys.

Indication of meteorologic conditions when sampling is active (i.e., "on effort", marked in green) and when sampling is non-active and considered opportunistic (i.e., "off effort", marked in red).

Sea State (Douglas scale)			Wind State (Beaufort scale)	
**Code**	**Height (m)**	**Description**	**Code**	**Description**
0	0	Calm (glassy)	0	Calm (oily/mirrored sea)
1	0 – 0.1	Calm (rippled)	1	Light air (smooth sea)
2	0.1 – 0.5	Smooth (wavelets)	2	Light breeze (looks like raining)
3	0.5 – 1.25	Slight	3	Gentle breeze (little white spots)
4	1.25 – 2.5	Moderate	4	Moderate breeze (several white spots)
5	2.5 – 4.0	Rough	5	Fresh breeze (white lines)
6	4.0 – 6.0	Very rough	6	Strong breeze (white lines w/ splashes)
7	6.0 – 9.0	High	7	Moderate gale (white lines w/ stretches)
8	9.0 – 14.0	Very high	8	Fresh gale
9	Over 14.0	Phenomenal	9	Strong gale
			10	Whole gale
			11	Storm
			12	Hurricane

Visibility (intuitive)		Rain	
**Code**	**Distance (m)**	**Code**	**Description**
1	< 50m	1	No rain
2	50 to 199 m	2	Little rain
3	200 to 499 m	3	Medium rain
4	500 to 999 m	4	Lots of rain
5	1000 to 1999m		
6	2000 to 3999 m		
7	4000 to 9 999 m		
8	10 000 to 19 999 m		
9	20 000 to 50 000 m		
10	> 50 000 m		

Supplementary Information S10. Code scripts.

Supplementary Information S10.1 Code script used for modelling the bias on the number of sightings recorded per survey under R software.

```
Libraries
library (foreign)
library (mgcv)
library (car)
library (boot)
library (pROC)
library (PerformanceAnalytics)
library (gratia)

Pearson Correlation
chart.Correlation(data, histogram=TRUE, method="pearson")

Variance Inflation Factor
vif(lm(Sight~Effort+Min_Sea+Max_Sea+Min_Wind+Max_Wind+Min_Vis+Max_Vis,
data=data))

Model Fitting
gam1<-
gam(Sight~s(Effort,k=4)+s(Min_Sea,k=4)+s(Max_Sea,k=4)+s(Min_Wind,k=4)+
s(Max_Wind,k=4)+s(Min_Vis,k=4)+s(Max_Vis,k=4),
family=poisson(link="log"),data = data)

Checking overfit
sum(residuals(gam1, type = "pearson")^2) / df.residual(gam1)
gamTh1<-
gam(Sight~s(Effort,k=4)+s(Min_Sea,k=4)+s(Max_Sea,k=4)+s(Min_Wind,k=4)+
s(Max_Wind,k=4)+s(Min_Vis,k=4)+s(Max_Vis,k=4), family=nb(),data =
data)
th<-gamTh1$family$getTheta(TRUE)

Backward selection
gam1<-
gam(Sight~s(Effort,k=4)+s(Min_Sea,k=4)+s(Max_Sea,k=4)+s(Min_Wind,k=4)+
s(Max_Wind,k=4)+s(Min_Vis,k=4)+s(Max_Vis,k=4),
family=negbin(theta=th),data = data)

gam2<-
gam(Sight~s(Effort,k=4)+s(Min_Sea,k=4)+s(Min_Wind,k=4)+s(Max_Wind,k=4)
+s(Min_Vis,k=4)+s(Max_Vis,k=4), family=negbin(theta=th),data = data)

AIC(gam1,gam2)
anova(gam1,gam2,test='Chisq')

gam3<-
gam(Sight~s(Effort,k=4)+s(Min_Sea,k=4)+s(Min_Wind,k=4)+s(Max_Wind,k=4)
+s(Max_Vis,k=4), family=negbin(theta=th),data = data)

AIC(gam3,gam2)
```

```
gam4<-
gam(Sight~s(Effort,k=4)+s(Min_Sea,k=4)+s(Min_Wind,k=4)+s(Max_Wind,k=4)
+s(Min_Vis,k=4), family=negbin(theta=th),data = data)

AIC(gam4,gam2)
anova(gam4,gam2,test='Chisq')

gam5<-
gam(Sight~s(Effort,k=4)+s(Min_Wind,k=4)+s(Max_Wind,k=4)+s(Min_Vis,k=4)
+s(Max_Vis,k=4), family=negbin(theta=th),data = data)

AIC(gam5,gam2)

gam6<-
gam(Sight~s(Effort,k=4)+s(Min_Sea,k=4)+s(Min_Wind,k=4)+s(Min_Vis,k=4)+
s(Max_Vis,k=4), family=negbin(theta=th),data = data)

AIC(gam6,gam2)

gam7<-
gam(Sight~s(Effort,k=4)+s(Min_Sea,k=4)+s(Max_Wind,k=4)+s(Min_Vis,k=4)+
s(Max_Vis,k=4), family=negbin(theta=th),data = data)

AIC(gam7,gam2)

gam8<-
gam(Sight~s(Min_Sea,k=4)+s(Min_Wind,k=4)+s(Max_Wind,k=4)+s(Min_Vis,k=4
)+s(Max_Vis,k=4), family=negbin(theta=th),data = data)

AIC(gam8,gam2)
```

Forward selection

```
gamA<-
gam(Sight~s(Effort,k=4)+s(Min_Sea,k=4)+s(Min_Wind,k=4)+s(Max_Wind,k=4)
+s(Min_Vis,k=4)+s(Max_Vis,k=4)+s(MEO,k=4)+s(LEO,k=4),
family=negbin(theta=th),data = data)

gamB<-
gam(Sight~s(Effort,k=4)+s(Min_Sea,k=4)+s(Min_Wind,k=4)+s(Max_Wind,k=4)
+s(Min_Vis,k=4)+s(Max_Vis,k=4)+s(Comb,k=4),
family=negbin(theta=th),data = data)

AIC(gamA,gamB)
anova(gamA,gamB,test='Chisq')

gamC<-
gam(Sight~s(Effort,k=4)+s(Min_Sea,k=4)+s(Min_Wind,k=4)+s(Max_Wind,k=4)
+s(Min_Vis,k=4)+s(Max_Vis,k=4)+s(Mean,k=4),
family=negbin(theta=th),data = data)

AIC(gamC,gamB)
anova(gamC,gamB,test='Chisq')
```

Backward selection

```
gamB1<-
gam(Sight~s(Effort,k=4)+s(Min_Sea,k=4)+s(Max_Sea,k=4)+s(Min_Wind,k=4)+
```

```
s(Max_Wind,k=4)+s(Min_Vis,k=4)+s(Max_Vis,k=4)+s(Cum,k=4),
family=negbin(theta=th),data = data)

gamB2<-
gam(Sight~s(Effort,k=4)+s(Min_Sea,k=4)+s(Min_Wind,k=4)+s(Max_Wind,k=4)
+s(Min_Vis,k=4)+s(Max_Vis,k=4)+s(Comb,k=4),
family=negbin(theta=th),data = data)

AIC(gamB1,gamB2)
anova(gamB1,gamB2,test='Chisq')

gamB3<-
gam(Sight~s(Effort,k=4)+s(Min_Sea,k=4)+s(Min_Wind,k=4)+s(Max_Wind,k=4)
+s(Max_Vis,k=4)+s(Comb,k=4), family=negbin(theta=th),data = data)

AIC(gamB3,gamB2)

gamB4<-
gam(Sight~s(Effort,k=4)+s(Min_Sea,k=4)+s(Min_Wind,k=4)+s(Max_Wind,k=4)
+s(Min_Vis,k=4)+s(Comb,k=4), family=negbin(theta=th),data = data)

AIC(gamB4,gamB2)
anova(gamB4,gamB2,test='Chisq')

gamB5<-
gam(Sight~s(Effort,k=4)+s(Min_Wind,k=4)+s(Max_Wind,k=4)+s(Min_Vis,k=4)
+s(Max_Vis,k=4)+s(Comb,k=4), family=negbin(theta=th),data = data)

AIC(gamB5,gamB2)

gamB6<-
gam(Sight~s(Effort,k=4)+s(Min_Sea,k=4)+s(Min_Wind,k=4)+s(Max_Wind,k=4)
+s(Min_Vis,k=4)+s(Max_Vis,k=4), family=negbin(theta=th),data = data)

AIC(gamB6,gamB2)

gamB7<-
gam(Sight~s(Effort,k=4)+s(Min_Sea,k=4)+s(Min_Wind,k=4)+s(Min_Vis,k=4)+
s(Max_Vis,k=4)+s(Comb,k=4), family=negbin(theta=th),data = data)

AIC(gamB7,gamB2)

gamB8<-
gam(Sight~s(Effort,k=4)+s(Min_Sea,k=4)+s(Max_Wind,k=4)+s(Min_Vis,k=4)+
s(Max_Vis,k=4)+s(Comb,k=4), family=negbin(theta=th),data = data)

AIC(gamB8,gamB2)

gamB9<-
gam(Sight~s(Min_Sea,k=4)+s(Min_Wind,k=4)+s(Max_Wind,k=4)+s(Min_Vis,k=4
)+s(Max_Vis,k=4)+s(Comb,k=4), family=negbin(theta=th),data = data)

AIC(gamB9,gamB2)

gamlin<-
gam(Sight~s(Effort,k=4)+s(Min_Sea,k=4)+Min_Wind+s(Max_Wind,k=4)+s(Min_
Vis,k=4)+Max_Vis+s(Comb,k=4), family=negbin(theta=th),data = data)
```

```
AIC(gamlin,gamB2)
anova(gamlin,gamB2,test='Chisq')
```

Best model

```
gam<-
gam(Sight~s(Effort,k=4)+s(Min_Sea,k=4)+Min_Wind+s(Max_Wind,k=4)+s(Min_
Vis,k=4)+Max_Vis+s(Cum,k=4), family=negbin(theta=th),data = data)
```

Diagnostic plots and adequacy of number of splines

```
gam.check(gam)
```

Influential data points

```
plot(influence.gam(gam))
```

Model residuals correlation

```
data1<-subset(data,Min_Wind!="NA")
summary(data1)

par(mfrow=c(3,3))
plot(data1$Effort,residuals(gam))
plot(data1$Min_Sea,residuals(gam))
plot(data1$Min_Wind,residuals(gam))
plot(data1$Max_Wind,residuals(gam))
plot(data1$Min_Vis,residuals(gam))
plot(data1$Max_Vis,residuals(gam))
plot(data1$Cum,residuals(gam))
```

Model final plots

```
gratia:::draw.gam(gam, parametric=TRUE, rug=TRUE, ci_col="#66CC99")

par(mfrow=c(3,3))
plot(gam, select = 1,xlab="Effort (Km)", ylab="s(Effort,
2.96)",ylim=c(-1.5,1.5),rug=T,cex.lab=1.25,cex.axis=1.25)
plot(gam, select = 2,xlab="Min Sea (Douglas Scale)", ylab="s(Min Sea,
2.15)",ylim=c(-0.8,0.2),rug=T,cex.lab=1.25,cex.axis=1.25)
termplot (gam,terms="Min_Wind", xlab=expression(paste("Min Wind
(Beaufort Scale)")), ylab="Partial for Min Wind",ylim=c(-1.5,0.1),
rug=T, se=T, col.term="black", lwd.term=1, col.res="black", pch=15,
cex=0.15, col.se="black",cex.lab=1.25,cex.axis=1.25)
plot(gam, select = 3,xlab="Max Wind (Beaufort Scale)", ylab="s(Max
Wind, 1.75)",ylim=c(-0.5,1),rug=T,cex.lab=1.25,cex.axis=1.25)
plot(gam, select = 4,xlab="Min Vis", ylab="s(Min Vis, 1.76)",ylim=c(-
0.5,0.3),rug=T,cex.lab=1.25,cex.axis=1.25)
termplot (gam,terms="Max_Vis", xlab=expression(paste("Max Vis")),
ylab="Partial for Max Vis",ylim=c(-2,0.3), rug=T, se=T,
col.term="black", lwd.term=1, col.res="black", pch=15, cex=0.15,
col.se="black",cex.lab=1.25,cex.axis=1.25)
plot(gam, select = 5,xlab="Combined Evaluation", ylab="s(Comb,
1.39)",ylim=c(-1,0.5),rug=T,cex.lab=1.25,cex.axis=1.25)
```

Supplementary Information S10.2 Code script used for modelling the bias on the identification success under R software.

```
Libraries
library (foreign)
library (mgcv)
library (car)
library(boot)
library(pROC)
library(PerformanceAnalytics)
library (gratia)

Character to Factor
class(data$Group)
data$Group<-as.factor(data$Group)

data1 <- data
data1[data1 == ""] <- NA
summary(data1)

Correlation
panel.cor <- function(x, y, digits=2, prefix="", cex.cor, ...)
{
  usr <- par("usr"); on.exit(par(usr))
  par(usr = c(0, 1, 0, 1))
  r <- abs(cor(x, y))
  txt <- format(c(r, 0.123456789), digits=digits)[1]
  txt <- paste(prefix, txt, sep="")
  if(missing(cex.cor)) cex.cor <- 0.8/strwidth(txt)
  text(0.5, 0.5, txt, cex = cex.cor * r)
}
pairs(~Size+Dist+Sea+Wind+Visibility+MEO+LEO+Cum+Mean, data=data1,
      lower.panel=panel.smooth, upper.panel=panel.cor,
      pch=20, na.action = na.omit, main="Variables Pair Plot Matrix")

Variance Inflation Factor
vif(lm(Success~Size+Dist+Sea+Wind+Visibility, data=data1))

Model Fitting

Backward selection
gam1 <-gam(Success ~ Group+s(Size,k=4)+s(Dist,
k=4)+s(Sea,k=4)+s(Wind,k=4)+s(Visibility,k=4),family = binomial(link =
"logit"), data = data1)

gam2 <-gam(Success ~ Group+s(Size,k=4)+s(Dist,
k=4)+s(Wind_state,k=4)+s(Visibility,k=4),family = binomial(link =
"logit"), data = data1)

AIC(gam1,gam2)
anova(gam1,gam2,test='Chisq')

gam3 <-gam(Success ~ Group+s(Size,k=4)+s(Dist,
k=4)+s(Wind_state,k=4),family = binomial(link = "logit"), data =
data1)
```

```
AIC(gam3,gam2)
anova(gam3,gam2,test='Chisq')

gam4 <-gam(Success ~ Group+s(Size,k=4)+s(Dist,
k=4)+s(Visibility,k=4),family = binomial(link = "logit"), data =
data1)

AIC(gam4,gam2)

gam5 <-gam(Success ~
s(Size,k=4)+s(Dist,k=4)+s(Wind_state,k=4)+s(Visibility,k=4),family =
binomial(link = "logit"), data = data1)

AIC(gam5,gam2)

gam6 <-gam(Success ~ Group+s(Size,
k=4)+s(Wind_state,k=4)+s(Visibility,k=4),family = binomial(link =
"logit"), data = data1)

AIC(gam6,gam2)

gam7 <-gam(Success ~ Group+s(Dist,
k=4)+s(Wind_state,k=4)+s(Visibility,k=4),family = binomial(link =
"logit"), data = data1)

AIC(gam7,gam2)
```

Forward selection

```
gamA <-gam(Success ~ Group+s(Size,k=4)+s(Dist,
k=4)+s(Wind,k=4)+s(Visibility,k=4)+s(MEO,k=4)+s(LEO,k=4),family =
binomial(link = "logit"), data = data1)

gamB <-gam(Success ~ Group+s(Size,k=4)+s(Dist,
k=4)+s(Wind_state,k=4)+s(Visibility,k=4)+s(Comb,k=4),family =
binomial(link = "logit"), data = data1)

AIC(gamA,gamB)
anova(gamB,gamA,test='Chisq')

gamC <-gam(Success ~ Group+s(Size,k=4)+s(Dist,
k=4)+s(Wind_state,k=4)+s(Visibility,k=4)+s(Mean,k=4),family =
binomial(link = "logit"), data = data1)

AIC(gamC,gamB)
anova(gamC,gamB,test='Chisq')
```

Backward selection

```
gamB1 <-gam(Success ~ Group+s(Size,k=4)+s(Dist,
k=4)+s(Sea,k=4)+s(Wind,k=4)+s(Visibility,k=4)+s(Cum,k=4),family =
binomial(link = "logit"), data = data1)

gamB2 <-gam(Success ~ Group+s(Size,k=4)+s(Dist,
k=4)+s(Sea_state,k=4)+s(Wind_state,k=4)+s(Visibility,k=4),family =
binomial(link = "logit"), data = data1)

AIC(gamB1,gamB2)
anova(gamB1,gamB2,test='Chisq')
```

```
gamB3 <-gam(Success ~ Group+s(Size,k=4)+s(Dist,
k=4)+s(Wind_state,k=4)+s(Visibility,k=4),family = binomial(link =
"logit"), data = data1)

AIC(gamB3,gamB2)
anova(gamB3,gamB2,test='Chisq')

gamB4 <-gam(Success ~ Group+s(Size,k=4)+s(Dist,
k=4)+s(Wind_state,k=4),family = binomial(link = "logit"), data =
data1)

AIC(gamB4,gamB3)

gamB5 <-gam(Success ~ Group+s(Size,k=4)+s(Dist,
k=4)+s(Visibility,k=4),family = binomial(link = "logit"), data =
data1)

AIC(gamB5,gamB3)

gamB6 <-gam(Success ~ s(Size,k=4)+s(Dist,
k=4)+s(Wind_state,k=4)+s(Visibility,k=4),family = binomial(link =
"logit"), data = data1)

AIC(gamB6,gamB3)

gamB7 <-gam(Success ~
Group+s(Size,k=4)+s(Wind_state,k=4)+s(Visibility,k=4),family =
binomial(link = "logit"), data = data1)

AIC(gamB7,gamB3)

gamB8 <-gam(Success ~ Group+s(Dist,
k=4)+s(Wind_state,k=4)+s(Visibility,k=4),family = binomial(link =
"logit"), data = data1)

AIC(gamB8,gamB3)
```

Best model

```
gam <-gam(Success ~ Group+s(Size,k=4)+s(Dist,
k=4)+s(Wind,k=4)+s(Visibility,k=4),family = binomial(link = "logit"),
data = data1)
```

Diagnostic plots and adequacy of number of splines

```
gam.check(gam)
```

Influential data points

```
plot(influence.gam(gam))
```

Model residuals correlation

```
data2<-subset(data1,Wind!="NA")
summary(data2)
nrow(data2)
data3<-subset(data2,Dist!="NA")
summary(data3)
data4<-subset(data3,Group!="NA")
summary(data4)
```

```
par(mfrow=c(3,2))
plot(data4$Group,residuals(gam))
plot(data4$Size,residuals(gam))
plot(data4$Dist,residuals(gam))
plot(data4$Wind,residuals(gam))
plot(data4$Visibility,residuals(gam))
```

Model final plots

```
gratia:::draw.gam(gam, parametric=TRUE, character=TRUE, rug=TRUE,
ci_col="#66CC99")

par(mfrow=c(2,3))
termplot (gam,terms="Group", xlab=expression(paste("Group")),
ylab="Partial for Group",ylim=c(-0.1,1), rug=T, se=T,
col.term="black", lwd.term=1, col.res="black", pch=15, cex=0.15,
col.se="black",cex.lab=1.25,cex.axis=1.25)
plot(gam, select = 1,xlab="Size", ylab="s(Size, 1.24)",ylim=c(-
2,30),rug=T,cex.lab=1.25,cex.axis=1.25)
plot(gam, select = 2,xlab="Distance (Scale 0-7)", ylab="s(Dist,
2.10)",ylim=c(-1.5,1),rug=T,cex.lab=1.25,cex.axis=1.25)
plot(gam, select = 3,xlab="Wind State (Beaufort Scale)", ylab="s(Wind
State, 2.31)",ylim=c(-0.25,1),rug=T,cex.lab=1.25,cex.axis=1.25)
plot(gam, select = 4,xlab="Visibility (Scale 0-10)",
ylab="s(Visibility, 2.34)",ylim=c(-
0.5,1),rug=T,cex.lab=1.25,cex.axis=1.25)
```
